# Regional homogeneity and functional connectivity of freezing of gait conversion in Parkinson’s disease

**DOI:** 10.3389/fnagi.2023.1179752

**Published:** 2023-07-12

**Authors:** Yiqing Bao, Yang Ya, Jing Liu, Chenchen Zhang, Erlei Wang, Guohua Fan

**Affiliations:** ^1^Department of Radiology, The Second Affiliated Hospital of Soochow University, Suzhou, China; ^2^Department of Neurology, The Second Affiliated Hospital of Soochow University, Suzhou, China

**Keywords:** Parkinson’s disease, regional homogeneity, freezing of gait, receiver operating characteristic curve, functional connectivity

## Abstract

**Background:**

Freezing of gait (FOG) is common in the late stage of Parkinson’s disease (PD), which can lead to disability and impacts the quality of life. Therefore, early recognition is crucial for therapeutic intervention. We aimed to explore the abnormal regional homogeneity (ReHo) and functional connectivity (FC) in FOG converters and evaluate their diagnostic values.

**Methods:**

The data downloaded from the Parkinson’s Disease Progression Markers Project (PPMI) cohort was subdivided into PD-FOG converters (*n* = 16) and non-converters (*n* = 17) based on whether FOG appeared during the 3-year follow-up; 16 healthy controls were well-matched. ReHo and FC analyses were used to explore the variations in spontaneous activity and interactions between significant regions among three groups of baseline data. Correlations between clinical variables and the altered ReHo values were assessed in FOG converter group. Last, logistic regression and receiver operating characteristic curve (ROC) were used to predict diagnostic value.

**Results:**

Compared with the non-converters, FOG converters had reduced ReHo in the bilateral medial superior frontal gyrus (SFGmed), which was negatively correlated with the postural instability and gait difficulty (PIGD) score. ReHo within left amygdala/olfactory cortex/putamen (AMYG/OLF/PUT) was decreased, which was correlated with anxiety and autonomic dysfunction. Also, increased ReHo in the left supplementary motor area/paracentral lobule was positively correlated with the rapid eye movement sleep behavior disorder screening questionnaire. FOG converters exhibited diminished FC in the basal ganglia, limbic area, and cognitive control cortex, as compared with non-converters. The prediction model combined ReHo of basal ganglia and limbic area, with PIGD score was the best predictor of FOG conversion.

**Conclusion:**

The current results suggested that abnormal ReHo and FC in the basal ganglia, limbic area, and cognitive control cortex may occur in the early stage of FOG. Basal ganglia and limbic area dysfunction combined with higher PIGD score are useful for the early recognition of FOG conversion.

## Introduction

Freezing of gait (FOG) is a distinct and serious gait impairment in the latter stage of Parkinson’s disease (PD) (Giladi et al., 1997, 2001), characterized by a sudden transient and unexpected interruption in walking, usually during gait initiation or while turning. PD patients with FOG frequently fall, which can lead to disability and impacts the quality of life (Perez-Lloret et al., 2014). Therefore, it is very important to explore the pathophysiological mechanisms of FOG. Understanding the determinants of future FOG can provide important prognostic information for clinicians.

In the last decade, numerous neuroimaging studies have reported abnormal structural and functional changes associated with FOG in PD patients. Gray matter loss and hypoperfusion were observed in various cortical regions, especially frontal and parietal cortices (Matsui et al., 2005; Kostic et al., 2012; Tessitore et al., 2012). Meanwhile, using functional MRI, several studies have reported that FOG may be associated with abnormal activation in the cortical cognitive control, sensorimotor networks and basal ganglia, as well as abnormal functional connections (FC) between them (Shine et al., 2013a,b; Canu et al., 2015; Gilat et al., 2018). Nonetheless, most previous studies included patients who already had FOG symptoms, and only a few MRI studies followed PD patients developing FOG over time to identify predictive brain signs of FOG conversion. Dadar and colleagues found that the amyloid-β pathology may be related to future FOG in PD patients through increasing the burden of white matter hyperintensities (Dadar et al., 2021). During FOG conversion, the left thalamus swell and FOG converters had a marked reduction in thalamo-cortical coupling with limbic and cognitive regions over the 2 years (D’Cruz et al., 2021). In a recent longitudinal study, Sarasso et al. (2022) compared the graph theory indices and clinical data of FOG converters, non-converters, and FOG patients at baseline and over a 2-year follow-up period, showing that over time FOG converters have more dyskinesia, executive dysfunction, and emotional disorders, as well as decreased parietal clustering coefficient and sensorimotor local efficiency. In summary, these previous studies provide evidence of structural and functional abnormities during FOG conversion, which may be used as imaging biomarkers to predict FOG.

In this study, using rest-state fMRI data, we aimed to combine regional homogeneity (ReHo), an index evaluating local signal synchronization by measuring the similarity between the time series of a chosen voxel and its adjacent voxels (Zang et al., 2004), with voxel-based functional connectivity (FC) analysis and clinical data to explore FOG conversion in PD patients from the Parkinson’s Disease Progression Markers Project (PPMI) cohort. Specifically, we used the second-year point-in-time data in PPMI cohort as baseline data and further subdivided it into FOG converters and non-converters based on whether FOG appeared during the following 3 years. Next, ReHo and FC analyses were performed in healthy controls and PD patients with and without FOG conversion to explore the changes of ReHo and FC in the early stage of FOG. Finally, logistic regression and characteristic curve (ROC) analysis were used to determine the best predictive model of FOG conversion with clinical data. Based on previous studies, we hypothesized that the alteration of ReHo and FC values in brain regions are specific to FOG converters in the early stage, and functional MRI combined with clinical features are most useful for the recognition of FOG conversion.

## Materials and methods

### Study design and participants

In this study, all data were used from the Parkinson’s Progression Markers Project (PPMI), a large-scale, comprehensive, multicenter Biomarker Project, which was committed to multiple clinical examinations, neuroimaging, and longitudinal follow-ups (Parkinson Progression Marker Initiative, 2011). Patients received a comprehensive evaluation in ON medication state. Since most PD patients did not collect resting-state functional MRI data in the first year of baseline, so we used data from the second year of follow-up as a baseline and assessed FOG conversion during the 3-year follow-up using MDS-UPDRS items 2.13 and 3.11. In the present study, the FOG converter was considered to be present if the score was ≥ 1 for either MDS-UPDRS item 2.13 or 3.11 at any point during the follow-up, and a persistent score of 0 was defined as the FOG non-converter (Kim et al., 2019; Dadar et al., 2021).

### Clinical assessments

Motor symptoms and disease severity were assessed using MDS-UPDRS III and HY stage. Based on MDS-UPDRS III, 11 items (2.10 and 3.15–18) were used for rest tremor score and 5 items (2.12–13 and 3.10–12) for postural instability and gait difficulty (PIGD) score (Stebbins et al., 2013; Dadar et al., 2021). Other clinical variables in this study included gender, duration, age, years of education, dominant hand, rapid eye movement (REM) sleep behavior disorder assessed by the REM Sleep Behavior Disorder Screening Questionnaire (RBDSQ), excessive daytime sleepiness assessed by the Epworth Sleepiness Scale (ESS), overall cognitive function assessed by the Montreal Cognitive Assessment (MoCA), depression assessed by the Geriatric Depression Scale Score (GDS), anxiety evaluated by the State-Trait Anxiety Inventory (STAI), and autonomic dysfunction evaluated by the Autonomic Outcome Scale in Parkinson’s disease (SCOPA-AUT). The PIGD, GDS, STAI, RBDSQ and SCOPA-AUT scores were sqrt-transformed (square root) to achieve a normal distribution.

### MRI data acquisition and preprocessing

T1-weighted gradient echo three-dimensional (3D) magnetization-prepared rapid gradient-echo (MPRAGE) sequences [repetition time (TR) = 2,300 ms, flip angle (FA) = 9°, and 1 mm^3^ isotropic voxels] were used to obtain high-resolution structural MRI images. Resting-state functional MRI scans were acquired with an echo-planar sequence (TR = 2,400 ms, FA = 80°, total of 210 volumes). The subjects were intended to close their eyes and rest and relax quietly. Functional images were preprocessed by data processing assistant for resting-state fMRI (DPARSF)^1^ and Statistical Parametric Mapping (SPM12)^2^ on the MATLAB r2018b platform as follows: (1) converting DICOM to NIFTI; (2) deleting the first 10 time points; (3) Slice timing correction; (4) Segmentation and realignment; (5) Regression of nuisance covariates (including white matter, cerebrospinal fluid, and Friston’s 24 parameters of head motion); (6) Spatial normalization to Montreal Neurological Institute (MNI) space by resampling to 3 mm × 3 mm × 3 mm by DARTEL; (7) Filtering (0.01 < f < 0.08 Hz). Subjects with maximal translations exceeding 3 mm or rotations > 3° were excluded from the statistical analysis. Additionally, the mean frame-wise displacement (FD) (Jenkinson et al., 2002) was calculated and added as a covariate in the statistical analysis.

### Statistical analysis

SPSS (Version 25.0. Armonk, NY, IBM Corp.) was used for clinical information analysis. Data normality was evaluated by the Shapiro–Wilk test. One-way analysis of variance, the Kruskal–Wallis test, two-sample *t*-test, or Wilcoxon Mann–Whitney test was utilized to compare the age, education experience, and clinical characteristics. Fisher’s exact test was employed for comparisons of categorical variables and component ratios. *P* < 0.05 was considered statistically significant.

The regions showing significant ReHo differences between FOG converters and non-converters were defined as regions of interest (ROI), which were chosen as the seeds for FC analysis. ReHo and FC data were spatially smoothed using a 6 mm full-width at half-maximum Gaussian kernel. An analysis of covariance (ANCOVA) was used to explore the ReHo and FC differences among the three groups with mean FD as the covariate. ReHo and FC results were, respectively corrected by AlphaSim (voxel *P* < 0.005 and cluster *P* < 0.01) and Gaussian random field correction (GRF) method (voxel level *P* < 0.001 and cluster level *P* < 0.05) for multiple comparisons. Effective ReHo and FC values of clusters with significant differences among groups were extracted, followed by *post-hoc* Bonferroni test, and ReHo values were correlated with clinical variables via Pearson’s correlation analysis.

Finally, the logistic regression model was used, including all MRI variables (i.e., ReHo and FC data) and clinical features with significant differences between groups at baseline. Univariate analysis was used to prove the influence of different factors on FOG conversion. We further excluded the possibility of collinearity and *P* > 0.01 factors in the univariable logistic analysis. Receiver operating characteristic (ROC) curve analysis was used to determine the best FOG prediction model.

## Results

### Demographic characteristics

A total of 17 FOG non-converters, 16 FOG converters, and 16 HCs were analyzed. The onset of PD patients included in our study was mostly laterality, with the right side being dominant in 63.6% of cases, and no difference was detected between the two groups with respect to age, gender, education year, dominant hand, and MoCA scores (*P* > 0.05). The rated values of H&Y, LEDD, ESS, GDS, RBDSQ, STAI, and tremor scores had no difference between converters and non-converters (*P* > 0.05), while MDS-UPDRS III, PIGD and SCOPA-AUT scores differed significantly across groups (*P* < 0.05) (Table 1).

**TABLE 1:** Demographic and clinical characteristics.

	FOG non-convertersg (*n* = 17)	FOG converters (*n* = 16)	HCs (*n* = 16)	*P*-value
Future FOG (Yes/No)	NO	YES	NA	<0.001
% Right-handed	88.2%	93.8%	87.5%	>0.05^a^
Age (years)	60.65 ± 9.33	63.34 ± 7.72	62.88 ± 9.03	0.640^b^
Gender (M/F)	11/6	11/5	13/3	0.624^a^
Education (year)	14.59 ± 3.02	15.75 ± 1.73	16.31 ± 1.85	0.161^b^
MoCA	28.00 (25.00, 30.00)	26.50 (24.25, 28.75)	28.00 (26.25, 28.75)	0.533^c^
Duration (months)	27.43 (26.23, 29.20)	27.40 (25.95, 29.88)	NA	0.885^d^
% Dominant side (right)	52.9%	75.0%	NA	0.141^a^
MDS-UPDRS III	15.76 ± 7.61	24.31 ± 11.03	NA	0.014^e^
PIGD	0.50 ± 0.55	1.24 ± 0.65	NA	0.001^e^
Tremor	4.82 ± 4.39	4.94 ± 4.28	NA	0.940^e^
H-Y stage	2.0 (1.0, 2.0)	2.0 (2.0, 2.0)	NA	0.090^d^
LEDD, mg	429.70 ± 222.07	505.70 ± 293.99	NA	0.407^e^
ESS	6.94 ± 3.91	8.13 ± 4.69	NA	0.436^e^
GDS	1.37 ± 0.87	1.54 ± 0.69	NA	0.543^e^
RBDSQ	1.89 ± 0.67	2.09 ± 0.82	NA	0.453^e^
STAI	8.04 ± 1.35	8.30 ± 0.97	NA	0.521^e^
SCOPA-AUT	2.98 ± 0.94	3.72 ± 0.97	NA	0.033^e^

HCs, healthy controls; MoCA, Montreal Cognitive Assessment; MDS-UPDRS, movement disorders society unified Parkinson’s Disease rating scale; FOG, freezing of gait; H&Y, hoehn and yahr stage; LEDD, levodopa equivalent daily dose; GDS, geriatric depression scale score; ESS, epworth sleepiness scale score; RBDSQ, rem sleep behavior disorder questionnaire score; STAI, State-Trait anxiety inventory; SCOPA-AUT, scale for outcomes in Parkinson’s Disease-Autonomic; Dominant side, side most affected at PD symptom onset; PIGD, postural instability and gait difficulty score; NA, not applicable. *P* < 0.05 was considered significant. ^*a*^By Fisher’s exact test. ^*b*^By One-way analysis of variance. ^*c*^By Kruskal-Wallis H test. ^*d*^By Mann-Whitney U test. ^*e*^By two-sample *t*-test.

### ReHo values

**FIGURE 1:**
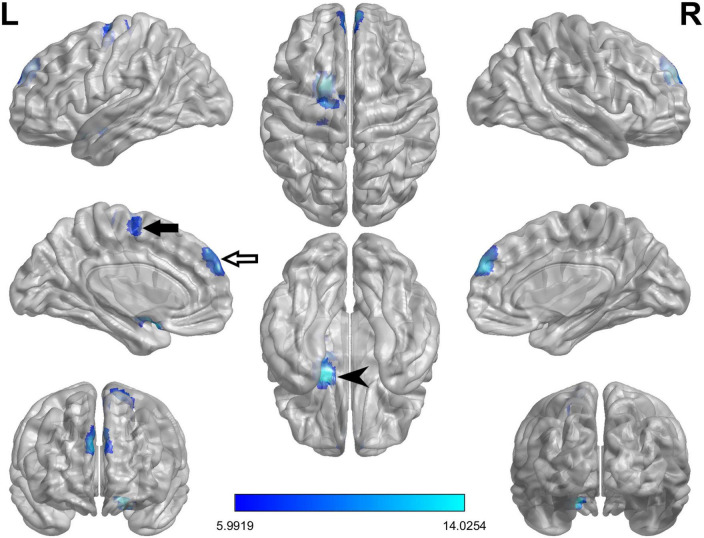
The ANCOVA test reveals significant differences in the ReHo index among FOG converter, FOG non-converter, and HC groups in the following regions: bilateral SFGmed (white arrow), left AMYG/PUT/OLF (black arrowhead) and left SMA/PCL (black arrow).

**FIGURE 2:**
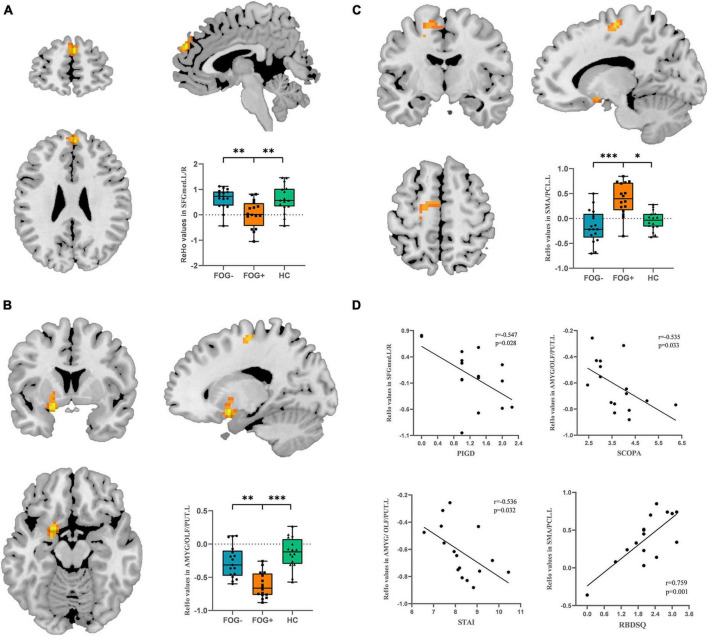
*Post hoc* analysis of ReHo differences. **(A)** Bilateral SFGmed demonstrated hypoactivity in FOG converters compared to FOG non-converter and HC groups. **(B)** Left OLF/PUT/AMYG demonstrated hypoactivity in FOG converters compared to FOG non-converter and HC groups. **(C)** Left SMA/PCL demonstrated significant hyperactivity in FOG converters compared to FOG non-converter and HC groups (Bonferroni corrected, *P < 0.05, ^**^P < 0.01, ^***^P < 0.001). **(D)** Correlation analysis between STAI, SCOPA scores and ReHo value in the left OLF/PUT/AMYG, RBDSQ and ReHo value in the left SMA/PCL (*P* < 0.05), PIGD score and ReHo value in the bilateral SFGmed. FOG+, FOG converters; FOG–, FOG non-converters.

The ReHo values of PD-FOG converter, non-converter, and HC groups differed significantly in the following locations: bilateral medial superior frontal gyrus (SFGmed), left amygdala/olfactory cortex/putamen (AMYG/OLF/PUT) and left supplementary motor area/paracentral lobule (SMA/PCL) (Table 2; Figure 1). Compared to PD-FOG non-converter group, the converter group showed decreased ReHo in the bilateral SFGmed, left OLF/PUT/AMYG (*P* < 0.01) (Table 2; Figures 2A, B), while increased ReHo in the left SMA/PCL (*P* < 0.001) (Table 2; Figure 2C). Compared to the HC group, the FOG converter group displayed decreased ReHo in the bilateral SFGmed (*P* < 0.01), left OLF/PUT/AMYG (*P* < 0.001) (Table 2; Figures 2A, B), while increased ReHo in the left SMA/PCL (*P* < 0.05) (Table 2; Figure 2C).

**TABLE 2:** ReHo differences among the three groups.

AAL regions	Number of voxels	Peak MNI coordinates			*F*-value
		*x*	*y*	*z*	
**Cluster 1**					
SFGmed.L	30	3	54	27	12.104
SFGmed.R	21				
**Cluster 2**					
SMA.L	15	−18	−12	60	10.055
PCL.L	9				
**Cluster 3**					
OLF.L	15	−18	6	−15	14.025
PUT.L	12				
AMYG.L	10				

SFGmed.L, left medial superior frontal gyrus; SFGmed.R, right medial superior frontal gyrus; SMA.L, left supplementary motor area, PCL.L, left paracentral lobule. OLF.L, left olfactory cortex; PUT.L, left putamen; AMYG.L, left amygdala. AAL, automated anatomical labeling.

### Correlation analysis

In PD-FOG converter group, the PIGD score was negatively correlated with the ReHo value in the bilateral SFGmed (*r* = −0.547, *P* = 0.028). Meanwhile, the ReHo value in the left OLF/PUT/AMYG was negatively correlated with SCOPA (*r* = −0.535, *P* = 0.033) and STAI scores (*r* = −0.536, *P* = 0.032). In contrast, a significantly positive correlation was found between the RBDSQ and the ReHo value in the left SMA/PCL (*r* = 0.759, *P* = 0.001) (Figure 2D).

### FC analysis

**FIGURE 3:**
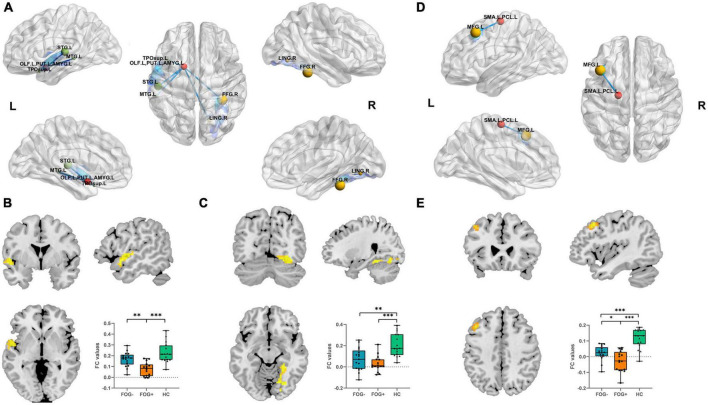
**(A)** Altered left OLF/PUT/AMYG FC value regions among three groups. **(B)** FC results among three groups with respect to left OLF/PUT/AMYG and left STG/MTG/TP/Rol. **(C)** FC results among the three groups within left OLF/PUT/AMYG and right FFG/LING. **(D)** Altered left SMA/PCL FC value regions among the three groups. **(E)** FC results among the three groups within left SMA/PCL and MFG (Bonferroni-corrected, *P < 0.05, ^**^P < 0.01, ^***^P < 0.001). FOG+, FOG converters; FOG–, FOG non-converters.

When the ROI was set to the left OLF/PUT/AMYG, three groups showed significantly different FC between left superior temporal gyrus/middle temporal gyrus/temporal pole/rolandic operculum (STG/MTG/TP/Rol), right fusiform gyrus/lingual gyrus (FFG/LING) and left OLF/PUT/AMYG (Table 3; Figure 3A). When the ROI was set to the left SMA/PCL, three groups showed significantly different FC between left middle frontal gyrus (MFG) and left SMA/PCL (Table 3; Figure 3D). When the ROI was set to the SFGmed, no cluster survived after GRF correction.

**TABLE 3:** FC differences among the three groups.

Brain regions (AAL)	Number of voxels	Peak MNI coordinates			*F*-value
		*x*	*y*	*z*	
**left SMA/PCL**					
MFG.L	47	−42	21	45	5.4072
**left OLF/PUT/AMYG**					
STG.L	68	−51	3	−6	3.9717
TPOsup.L	15				
MTG.L	14				
Rol.L	3				
FFG.R	89	27	−45	−12	3.8388
LING.R	25				

SMA, supplementary motor area; PCL, paracentral lobule; OLF, olfactory cortex; PUT, putamen; AMYG, amygdala; MFG.L, left middle frontal gyrus; STG.L, left superior temporal gyrus; TPOsup.L, left temporal pole (TP): superior temporal gyrus; MTG.L, left middle temporal gyrus; Rol.L, left rolandic operculum, FFG.R, right fusiform gyrus; LING.R, right lingual gyrus. AAL, automated anatomical labeling.

Compared to FOG non-converter group, the converter group showed decreased FC between left STG/MTG/TP/Rol (*P* < 0.01) (Table 3; Figure 3B) and left OLF/PUT/AMYG, left MFG (*P* < 0.05) and left SMA/PCL (Table 3; Figure 3E).

Compared to HC group, the FOG converter group showed decreased FC between left STG/MTG/TP/Rol (*P* < 0.001) (Table 3; Figure 3B), right FFG/LING (*P* < 0.001) (Table 3; Figure 3C) and left OLF/PUT/AMYG, left MFG (*P* < 0.001) and left SMA/PCL (Table 3; Figure 3E).

Compared to HC group, the FOG non-converter group showed decreased FC between right FFG/LING (*P* < 0.01) (Table 3; Figure 3C) and left OLF/PUT/AMYG, left MFG (*P* < 0.001) and left SMA/PCL (Table 3; Figure 3E).

### ReHo and FC data as predictors of FOG

The results of the logistic regression analyses are presented in Table 4. In the univariable logistic regression analyses, the decreased ReHo value in the SFGmed [odds ratio (OR): 0.76, 95% confidence interval (CI): 0.62–0.93, *P* = 0.007], left OLF/PUT/AMYG (OR: 0.46, 95% CI: 0.27–0.78, *P* = 0.004) and the increased ReHo value in the left SMA (OR: 1.61, 95% CI: 1.17–2.22, *P* = 0.003) were significantly associated with FOG conversion. Decreased FC between left OLF/PUT/AMYG and left STG/MTG/TP/Rol (OR: 0.11, 95% CI: 0.03–0.49, *P* = 0.004), left SMA/PCL and left MFG (OR: 0.18, 95% CI: 0.04–0.80, *P* = 0.024) were related to FOG conversion. In addition, MDS-UDPRS III (OR: 1.01, 95% CI: 1.00–1.02, *P* = 0.025), PIGD (OR: 1.23, 95% CI: 1.06–1.42, *P* = 0.007), and SCOPA-AUT score (OR: 1.09, 95% CI: 1.00–1.20, *P* = 0.047) were predictive of FOG conversion.

**TABLE 4:** Logistic regression analysis of clinical and MR data.

Variables	Univariate analysis		Multivariate analysis	
	OR (95% CI)	*P*	OR (95% CI)	*P*
	OR (95% CI)	P	OR (95% CI)	P
ReHo^1^	0.76 (0.62–0.93)	0.007		
ReHo^2^	0.46 (0.27–0.78)	0.004	0.39 (0.18–0.85)	0.017
ReHo^3^	1.61 (1.17–2.22)	0.003		
FC^1^	0.11 (0.03–0.49)	0.004		
FC^2^	0.18 (0.04–0.80)	0.024		
MDS-UPDRS III	1.01 (1.00–1.02)	0.025		
PIGD	1.23 (1.06–1.42)	0.007	1.31 (1.04–1.64)	0.021
SCOPA	1.09 (1.00–1.20)	0.047		
Tremor	1.00 (0.99–1.02)	0.938		
GDS	1.03 (0.94–1.13)	0.531		
RBDSQ	1.04 (0.94–1.14)	0.441		
STAI	1.02 (0.96–1.08)	0.510		

ReHo^1^, ReHo value in bilateral SFGmed; ReHo^2^, ReHo value in left OLF/PUT/AMYG; ReHo^3^, ReHo value in left SMA/PCL; FC^1^, FC value between left OLF/PUT/AMYG and left STG/MTG/TP/Rol; FC^2^, FC value between left SMA/PCL and MFG. *P* < 0.05 was considered significant.

### Predictive value of clinical and ReHo data

**FIGURE 4:**
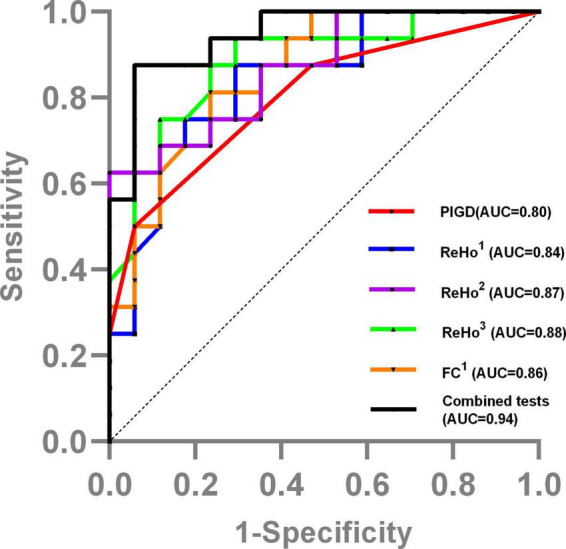
The ROC curve showed the value of clinical and MRI data in distinguishing PD-FOG converters and non-converters. ReHo^1^, ReHo value in bilateral SFGmed; ReHo^2^, ReHo value in left OLF/PUT/AMYG; ReHo^3^, ReHo value in left SMA/PCL; FC^1^, FC value between left OLF/PUT/AMYG and left STG/MTG/TP/Rol; Combined tests, the tests combined the PIGD score with the ReHo value in basal ganglia and limbic area.

Stepwise selected by logistic regression, the final best prediction model for FOG conversion included PIGD score (OR: 1.31, 95% CI: 1.04–1.64, *P* = 0.021) and ReHo value in basal ganglia and limbic area (i.e., left OLF/PUT/AMYG) (OR: 0.39, 95% CI: 0.18–0.85, *P* = 0.017). The ROC curve analysis showed that the combined model of ReHo value and clinical variable distinguished between PD-FOG converters and non-converters [area under curve (AUC) = 0.94, 95% CI: 0.87–1], with a sensitivity of 87.5% and specificity of 94.1% (Figure 4).

## Discussion

In this study, we analyzed the differences in MRI variables and clinical features of FOG converters and non-converters in PD patients, as well as their values in identifying FOG converters at the follow-up. The current results supported that cognitive control cortex, basal ganglia, and limbic area dysfunction may be related to PD-FOG conversion, and the decreased ReHo value in basal ganglia and limbic area, combined with PIGD score is the best initial predictive model.

Significantly decreased ReHo value within left putamen in the FOG converter group may suggest that such regional impairment contributes to the pathophysiology of FOG conversion in this study. In PD patients, several studies (Goldstein et al., 2017; Kish et al., 2017) have reported dopamine deficiency in the striatum and found that the most severely affected region was putamen. The putamen was suggested to be involved in self-initiated movement and its dysfunction was shown to relate to bradykinesia (Rodriguez-Oroz et al., 2009; Prodoehl et al., 2010; Dirnberger and Jahanshahi, 2013). A positron emission tomography (PET) study has also reported a relationship between FOG and dysfunction within the putamen; FOG was correlated with reduced F-Dopa uptake in the putamen of the right hemisphere (Pikstra et al., 2016). FOG converters also showed increased ReHo value in the left SMA/PCL and decreased FC between left SMA/PCL and MFG compared to non-converters. MFG, a critical brain region of the cognitive control network, is responsible for executive functions in the cognitive control network (MacDonald et al., 2000), while SMA plays a crucial role in the early preparation of voluntary movements (Cunnington et al., 2002). Hence, we assumed that neural activation can be expected in cortical motor regions as a compensation strategy for basal ganglia dysfunction and abnormal functional connectivity in the prefrontal cortex (Sabatini et al., 2000; Eckert et al., 2006; Redgrave et al., 2010). These findings were in keeping with the decreased activation of putamen and increased activation of cortical motor regions in FOG during motor block (Wu and Hallett, 2005; Vercruysse et al., 2014), as insufficient compensatory brain activity which might result in difficulties in stride length regulation, ultimately leading to FOG. In contrast to our findings, Zhou et al. (2018) demonstrated that PD-FOG patients had reduced ReHo value in the left SMA. We consider it might be due to the inadequate compensatory ability of the motor cortex in the late stage of FOG; it may also be due to the formation of indirect basal ganglia pathways leading to subcortical overactivation, thus inhibiting cortical activity. Furthermore, we also discovered that RBDSQ was positively correlated with the ReHo value of left SMA in FOG converters. Similar to our results, during REM sleep, compared to the eyes closed awake condition, activity was higher in SMA in a magnetoencephalographic tomography (Ioannides et al., 2004). One reason for this could be the significant overlap between functional neuroanatomy that controls REM sleep, arousal and locomotion as well as multiple neural circuits affected in RBD and PD with FOG. However, the pathophysiology between REM and FOG also remains unclear; thus, more research is needed in this area.

The current findings indicated that the dysfunction in limbic brain region may be a risk factor for FOG conversion. Olfactory cortex is a significant part of the limbic system. Microstructural white matter reductions are present in the central olfactory system of early-stage PD patients (Ibarretxe-Bilbao et al., 2010), and additionally compared to PD patients without FOG, FOG patients had more severe olfactory deficits (Glover et al., 2021). In Alzheimer’s disease, olfaction is a strong predictor of cognitive decline, with atrophy in the amygdala, hippocampus, and olfactory cortex (Vasavada et al., 2015; MacDonald et al., 2018; Lian et al., 2019). In this study, the olfactory cortex in FOG converters exhibited a reduced ReHo expression compared to non-converters, which may be related to the production of cognitive load and a decline in attention control. Decreased FC of left basal ganglia and limbic area with left temporal cortex including left STG, MTG, and TP was observed in this study as well. Included in the default-mode network (DMN), the temporal lobe is related to memory, visual-spatial ability, and the ability to read the social attention cues of others (Rektorova et al., 2014). Mild cognitive impairment (MCI) patients had atrophy and reduced ALFF value of the temporal cortex (Pereira et al., 2012; Gao et al., 2016). This result is attributed to a link between cognitive impairment and degeneration or inactivation in the temporal gyrus. FOG patients seem to have difficulty in execution and acquisition of automation compared to non-FOG patients (Hackney and Earhart, 2010; Spildooren et al., 2010; Vandenbossche et al., 2013). Loss of automation indicated significant pressure on cognitive resources (Vandenbossche et al., 2012). Perhaps, this might explain that FOG patients use the same attention resources to control their mood. Thus, due to executive dysfunction, FOG episodes occur when cognitive resources overload (Eysenck et al., 2007). Furthermore, served as the subcortical hub of the limbic system, amygdala controlled both the emotional behavior and the autonomic nervous system (Ulrich-Lai and Herman, 2009). Previous studies have shown that there were higher heart rates and gastrointestinal autonomic dysfunction during FOG episodes (Maidan et al., 2010; Kwon et al., 2021). Meanwhile, emotional states have been shown to affect the motor control of gait (Naugle et al., 2011; Kyrdalen et al., 2019), and volume atrophy and hypoactivation in the amygdala have been found associated with major depressive disorder (Xia et al., 2004; Sheng et al., 2014; Li et al., 2022). Our study further supports the correlation between amygdala dysfunction and FOG conversion.

Notably, our findings suggested that FOG converters exhibited a negative correlation between the PIGD score and decreased ReHo value in the SFGmed. SFGmed belongs to the medial prefrontal lobe (mPFC) and has differential associations with executive function and attention allocation, value-based decision-making, and emotion regulation (DiGirolamo et al., 2001; Rushworth et al., 2002; Ragozzino, 2007). Hypoperfusion and reduced activation in bilateral mPFC has been reported in PD patients with difficulty initiating motor movement (Playford et al., 1992; Matsui et al., 2005). Gray matter atrophy in the mPFC has also been observed in PD patients with dyskinesia (Dagan et al., 2017; Zheng et al., 2022). Thus, dysfunction in mPFC could help reveal the neural pathophysiology related to the FOG. In our study, with no significant difference between the two study groups, FOG converters had higher LEDD and non-motor symptom scores; combined with higher PIGD scores, this result may indicate a low response to medication in FOG converters. PD patients with higher PIGD scores also had more severe motor and non-motor symptoms, rapid disease progression, and poorer experiences of daily living (Jankovic et al., 1990; Rajput et al., 2009); these phenomena might be due to the formation of Lewy bodies and significant amyloid plaque load in the mPFC (Selikhova et al., 2009).

This study has the significant advantage of combining MRI variables and clinical features, suggesting that basal ganglia and limbic area dysfunction combined with a higher PIGD score resulted in the highest predictive model accuracy (AUC = 0.94). Although with valuable findings, there are still some limitations in this study. First, the incidence of PD patients included in our study is mainly on the right side, which may be the reason why most abnormal brain regions and functional connections occurred on the left side. Second, there are a limited number of participants in our study. Therefore, more variables could not be added to the regression model, and thus, our findings should be interpreted with caution. In addition, we lacked a detailed gait assessment, such as gait speed, stride length, and other features to assess the motor status, which provided limited clinical data. However, our above study is prospective, and the results are similar to previous studies. Thus, our current grouping and sample size can explain the results, but the conclusions require a larger sample size and resampling by replication to eliminate the confounding factors and further explore their authenticity.

## Conclusion

The current results supported that functional MRI could be considered a useful tool to identify FOG conversion in PD: the basal ganglia and limbic area dysfunction could represent a marker of FOG conversion, together with the PIGD score. The important role of functional MRI to monitor disease conversion and to detect brain changes might precede the conversion of specific clinical features such as FOG. Thus, we hope that these abnormalities lay a foundation for improvement in early clinical early screening.

## Data availability statement

The original contributions presented in this study are included in the article/supplementary material, further inquiries can be directed to the corresponding authors.

## Ethics statement

Written informed consent was obtained from the individual(s) for the publication of any potentially identifiable images or data included in this article.

## Author contributions

YB analyzed the data, plotted tables, and wrote the manuscript. YY and CZ re-analyzed the data and checked the figures. JL provided us with clinical scale analyses. EW and GF designed and organized the study. All authors contributed to the manuscript revision and read and approved the submitted version.

## Footnotes

1
http://www.restfmri.net/forum/dparsf

2
http://www.fil.ion.ucl.ac.uk/spm
